# Modeling Network-Controlled Device-to-Device Communications in SimuLTE

**DOI:** 10.3390/s18103551

**Published:** 2018-10-19

**Authors:** Giovanni Nardini, Antonio Virdis, Giovanni Stea

**Affiliations:** Dipartimento di Ingegneria dell’Informazione, University of Pisa, 56122 Pisa, Italy; g.nardini@ing.unipi.it (G.N.); antonio.virdis@unipi.it (A.V.)

**Keywords:** simulation, LTE, direct communication, D2D, SimuLTE

## Abstract

In Long Term Evolution-Advanced (LTE-A), network-controlled device-to-device (D2D) communications allow User Equipments (UEs) to communicate directly, without involving the Evolved Node-B in data relaying, while the latter still retains control of resource allocation. The above paradigm allows reduced latencies for the UEs and increased resource efficiency for the network operator, and is therefore foreseen to support several services, from Machine-to-machine to vehicular communications. D2D communications introduce research challenges that might affect the performance of applications and upper-layer protocols, hence simulations represent a valuable tool for evaluating these aspects. However, simulating D2D features might pose additional computational burden to the simulation environment. To this aim, a careful modeling is required to reduce computational overhead. In this paper, we describe our modeling of network-controlled D2D communications in SimuLTE, a system-level LTE-A simulation library based on OMNeT++. We describe the core modeling choices of SimuLTE, and show how these allow an easy extension to D2D communications. Moreover, we describe in detail the modeling of specific problems arising with D2D communications, such as scheduling with frequency reuse, connection mode switching and broadcast transmission. We document the computational efficiency of our modeling choices, showing that simulation of D2D communications is not more complex than simulation of classical cellular communications of comparable scale. Results show that the heaviest computational burden of D2D communication lies in estimating the Sidelink channel quality. We show that SimuLTE allows one to evaluate the interplay between D2D communication and end-to-end performance of UDP- and TCP-based services. Moreover, we assess the accuracy of using a binary interference model for frequency reuse, and we evaluate the trade-off between speed of execution and accuracy in modeling the reception probability.

## 1. Introduction

The now ubiquitous diffusion of 4G cellular networks, under the commercial names of Long Term Evolution (LTE) and LTE-Advanced (LTE-A), makes them ideal candidates to support new scenarios such as Smart Cities, Connected Vehicles, Machine-to-machine (M2M) communications [1] and Industry 4.0. LTE-A runs on licensed spectrum, which is therefore reliable, has plug-and-play capabilities, built-in security features and QoS mechanisms. It is also foreseeable that the upcoming 5G standards will embody the basic design principles of LTE-A, evolving them to fit the new paradigm. A feature that has appeared in the latest LTE-A standards, which also represents a pillar of current 5G-oriented research, is device-to-device (D2D) communications, whereby two User Equipments (UEs) can communicate directly, without their traffic being relayed by the evolved Node-B (eNB) as in traditional cellular communications. The benefits of D2D communications are manifold, for both the network operator and the user. From an operator standpoint, D2D transmissions can be supervised by the eNB, in a network-controlled approach: the eNB remains in charge of the scheduling and resource allocation, but cuts itself off from data-plane transmission. This makes D2D transmissions reliable, unlike those in ad-hoc networks, where single mobiles have to contend for spectrum access through carrier sensing, collisions and backoffs. This very feature allows the eNB to enforce frequency reuse, allowing simultaneous D2D transmissions to occur on the same frequency resources, provided that the respective receivers are distant enough, which enhances scalability. From a user standpoint, D2D transmissions are normally faster, since they require a single hop instead of two (i.e., uplink first to the serving eNB, and downlink next to the receiving UE). Moreover, they enable proximity services, i.e., services where the endpoints of the communications must be in geographical proximity. Proximity services include those for IoT transmissions (e.g., [2,3]) and those for vehicular systems (e.g., for platooning [4,5] or collision alert [6]). A different paradigm, still based on D2D communications, is that of network-unassisted communications, whereby LTE-A UEs setup either point-to-point or point-to-multipoint communications without the help of the eNB. This is useful, for instance, in public-safety scenarios, where UEs can setup an emergency infrastructureless network, or to extend network coverage using UEs as relays [7]. The main issues with network-unassisted D2D, such as device discovery and synchronization, are different from those of network-controlled D2D, such as frequency reuse and mode selection. This paper deals with network-controlled D2D communications (henceforth, D2D communications).

D2D communications introduce several challenges that have been studied by the research community [8]. However, evaluating services based on D2D communications in LTE-A is a challenging task. To date, few testbeds, if any, have been made available to the public. In any case, testbeds are unlikely to scale to the levels required to test these systems effectively (i.e., with interference from competing non-D2D transmissions, across multiple cells, and in mobility). On the other hand, link-level simulators (e.g., [9]) are designed for different purposes: they model the channel—specifically, the D2D channel or sidelink (SL)—sometimes to a great level of accuracy, but they neglect the upper layers of the system, which—as we show in this paper—play a crucial role in the performance of such services.

In this paper, we show how D2D communications are modeled in SimuLTE, an open-source simulation library for system-level simulation of LTE/LTE-A cellular networks based on OMNeT++, which is widely used in the research community [10,11]. By simulation library, we mean a collection of models with well-defined interfaces, which can be instantiated and connected to build arbitrarily complex simulation scenarios. It is based on the OMNeT++ framework [12], and is fully compatible with INET [13], another OMNeT++-based library that simulates standard Internet entities. We first provide a general outlook on SimuLTE, discussing the relevant modeling choices and their impact on performance, and then explain the modeling of D2D communications, and in particular: how to modify the LTE protocol stack to accommodate the coexistence of traditional and D2D communications, modeling resource allocation with frequency reuse, and message transmission. We then discuss profiling results of SimuLTE execution, assessing the impact of D2D communications on the simulator performance, and then show examples of simulations employing D2D. To the best of our knowledge, SimuLTE is the only simulation library modeling network-controlled D2D communications. There are two other major system-level simulation libraries, i.e., LENA for ns3 [14] and LTESim [15]. LENA has a D2D add-on [16] that deals with network-unassisted D2D communications, whereas LTESim does not seem to support D2D.

The main contributions of the paper can be summarized as follows:

- The documentation of an efficient modeling of D2D communications for the widely used SimuLTE system-level simulator that allows researchers to validate the modeling and to extend the tool for their own research purposes;
- A discussion of the performance of the tool, in terms of complexity and profiling of execution time;
- Exemplary results showing the type of scenarios that can be run involving D2D communications, and the type of insight that can be gained by system-level simulation; and
- An assessment of the effects of parameter selection on the observable performance (namely, interference radius vs. frequency reuse, transmission horizon vs. reception probability and execution speed in multihop D2D communications).

The rest of the paper is organized as follows: Section 2 introduces the relevant background on the LTE-A technology. In Section 3, we review the related works in detail. Section 4 describes the general architecture of SimuLTE, whereas Section 5 discusses the modeling of D2D communications within it. Section 6 reports evaluation results, and Section 7 concludes the paper.

## 2. Background on LTE-Advanced

This section provides the necessary background on LTE-A and introduces the related terminology and abbreviations. We limit our description to those features that are relevant to D2D communications, namely protocol layering and resource allocation. At the end of this section, we introduce network-controlled D2D communications in LTE-A.

**Figure 1:**
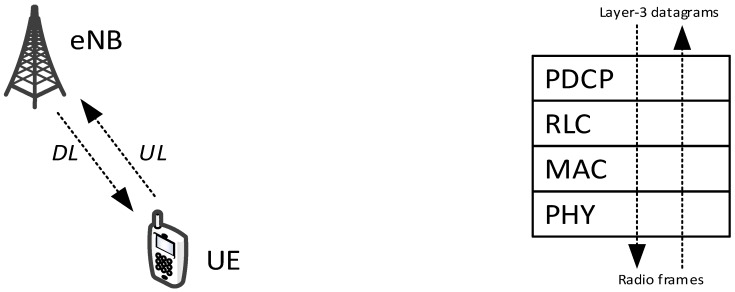
LTE-A radio access network (**left**); and protocol layering (**right**).

The radio access network of LTE-A is depicted in Figure 1 (left). It is composed of UEs and base stations, called eNBs in the LTE terminology. At any time, a UE is connected to one eNB and sends/receives radio frames to/from it through the uplink (UL) and downlink (DL), respectively.

LTE-A is a layer-2 technology. However, the LTE-A protocol stack consists of four distinct sublayers, as shown in Figure 1 (right). An IP packet sent down to the LTE-A interface is first handled by the Packet Data Convergence Protocol (PDCP). The latter creates the PDCP context for the (flow of) packet(s), i.e., ciphers the packet with a ciphering key, so that the latter can be deciphered only at the PDCP layer of the intended recipient, and assigns to it a progressive sequence number valid within that context. The packet is then passed down to the Radio Link Control (RLC) layer, in the form of an RLC Service Data Unit (SDU). The RLC can be configured in one of three modes: transparent (TM), unacknowledged (UM) and acknowledged (AM). The RLC TM adds nothing. The RLC UM, which is the one recommended by the standard for D2D, performs segmentation/concatenation of RLC SDUs on transmission, and reassembly, duplicate detection and reordering of RLC PDUs on reception. The RLC AM adds control messages (such as RLC STATUS PDUs) flowing in the opposite direction to confirm reception at the RLC level. RLC SDUs are buffered at the RLC. The underlying MAC fetches data from the RLC buffers, by requesting to it an RLC Protocol Data Unit (PDU) of a given size. The RLC complies by dequeueing from its buffer one or more (fragments of) RLC SDUs and combining them as necessary into RLC PDUs. The MAC adds its own header and forms a MAC PDU, also called Transmission Block (TB), which is then sent to the physical (PHY) layer for transmission over the radio interface.

Access to the physical layer is regulated by the eNB, which decides which frequency resources are allocated to which transmission. In LTE-A, UL and DL transmissions occur periodically, at 1 ms intervals called Transmission Time Intervals (TTI). With reference to a Frequency-division Duplexing (FDD) deployment, the UL and DL subframes are transmitted on non-overlapping frequency bands on every TTI. In the frequency domain, a subframe can be seen as a logical vector of Resource Blocks (RBs). A TB occupies an integer number of RBs.

**Figure 2:**
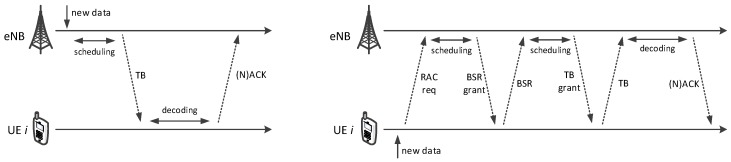
Procedures for DL (**left**) and UL (**right**) data transmissions.

DL transmissions carry traffic buffered at the eNB to the UEs (Figure 2, left). The eNB selects the modulation and coding to be used (which determines how many information bits will fit in a RB), based on the Channel Quality Indicator (CQI) reported by the UE, which reflects its measured Signal to Interference and Noise Ratio (SINR). A Hybrid ARQ (H-ARQ) scheme, which allows a configurable number of retransmissions, provides reliability at the MAC layer: the receiving UE sends back either an ACK or a NACK four TTIs after the transmission. The eNB may then reschedule a retransmission at any future TTI (DL H-ARQ is asynchronous). The Orthogonal Frequency-division Multiple Access (OFDMA) scheme is used for physical transmissions in the DL.

UL subframes carry UE traffic destined to the eNB (Figure 2, right). The latter issues transmission grants to the UEs, specifying on which RBs they can send their TB, using what transmission format. Single-Carrier Frequency-division Multiple Access (SC-FDMA) scheme is used for transmissions. Since UL buffers reside at the UEs, UEs must signal their backlog to the eNB through Buffer Status Reports (BSRs). BSRs are sent in band, possibly trailing a data transmission, whenever the UE is scheduled and has enough space to do so (a BSR can take up to 24 bits). New backlog for a previously idle UE can instead be signaled through an out-of-band Random-access (RAC) procedure. RAC requests are contention-based, and the eNB responds by scheduling the UE in a future TTI. Unanswered RAC requests are re-iterated. H-ARQ protects UL transmissions as well, and it works like the DL one, the main difference being that it is synchronous: the eNB schedules UL retransmissions after a constant lag of eight TTI.

### Network-Controlled Device-to-Device Communications

Network-controlled D2D communications have been added to the LTE-A standard in release 12 [17]. The standard deals with point-to-multipoint (P2MP), or one-to-many, communications. The SL is often physically allocated in the UL spectrum in an FDD deployment, since UL frequencies are less likely to be congested than DL ones. This requires D2D-enabled UEs to be able to receive SC-FDMA transmissions besides OFDMA ones [7]. Resource allocation on the SL is done by the eNB, and it can be either static or on demand. In a static approach, called Autonomous Resource Selection (ARS), the eNB configures a static resource pool, e.g., *k* RBs every *T* TTIs, and UEs can use these resources without any signaling. This implies some latency, since a UE must wait for the next TTI where resources are available, and does not regulate collisions, since two nearby UEs may decide to use the same resources simultaneously. The Scheduled Resource Allocation (SRA), on the other hand, is an on-demand scheme which mirrors resource allocation in the UL for standard communications: the UE sends a Random Access (RAC) request to the eNB, which grants enough space for it to send its BSR. Then, the eNB schedules SL resources accordingly and issues the grant to the UE for D2D communications. SRA allows the eNB to schedule interference-free transmissions, at the price of more control traffic.

P2MP D2D transmissions are sent as multicast MAC-layer transmissions. This is done by using a multicast group ID as a MAC ID in the message. They are heard by whichever UE is within hearing distance of the transmitter, and they are not acknowledged. Therefore, a sender has no way to know which neighboring UEs, if any, actually received a message.

Point-to-point (P2P) or unicast D2D communications have not been standardized yet. However, there is a large body of scientific literature on the subject, which substantially agrees on what the transmission paradigm should be: with P2P D2D, a sender UE *a* can send a packet to one (and only one) receiver UE *b*, when the eNB allows them to use the SL. This is done by granting *a* one or more RBs for transmission and instructing *b* to listen on the same RBs for reception, still assuming that *b* is equipped with a SC-FDMA receiver. UEs *a* and *b* form a unidirectional flow, whose data are not relayed at the eNB. A key requirement of P2P D2D communications is that they should be transparent to the upper layers, so that the decision to route a flow to the SL or through the traditional UL/DL cellular data path can be taken dynamically by the eNB, without the sender/receiver applications being aware of it (save, possibly, for experiencing different performance in the two modes). The above decision, called mode selection, may be taken according to different policies (channel quality on the UL/DL/SL, congestion, possibility of frequency reuse, etc.), and it entails a set of non-trivial decisions to be made at the two endpoints and the eNB—e.g., whether to drop or reschedule buffered traffic—which go by the name of mode switching (MS) (see [18] for a comprehensive discussion of the subject).

The above discussion assumed that D2D communications are arranged in licensed spectrum. However, D2D communications can also occur in unlicensed spectrum. The latter can be added to the licensed one to increase the capacity of the system. In such deployments, additional mechanisms for managing the interference with other wireless technologies (e.g., Wi-Fi) must be put in place [19,20,21]. In this paper, we focus on D2D communications in the licensed spectrum only.

## 3. Related Work

LTE simulation tools can be divided into link-level and system-level. As already anticipated, link-level tools deal with the physical aspects of transmissions and reception, but do not model the upper layers (they often stop at the MAC layer of LTE). A notable example is the Vienna LTE simulator [22], developed in Matlab, and used by a large community. The work in [8] adds D2D capabilities to it. The work in [23] presents a standalone simulator for the DL of LTE-A. In the latter, written in C++, physical-layer modeling is given great consideration, and the MAC layer is also modeled. However, packets are assumed to be generated at the RLC according to pre-defined patterns (e.g., full-buffer, streaming). The work in [24] presents LTEV2Vsim, a Matlab-based standalone simulator for Vehicle-to-vehicle (V2V) communications. It includes vehicle mobility and models LTE-based communication, under the form of transmission/reception of beacons for vehicles. Some standalone simulators meant to assess D2D performance in LTE have been released in the past few years [25,26]. In [25], the authors developed scenarios to simulate the discovery of potential D2D peers, based on physical-layer conditions (no packets are transmitted between users). The work in [26] describes a simulator where UEs move according to a random waypoint model in a multicell environment, and assesses the physical interference between UL and D2D transmissions.

The above link-level simulators cannot support simulations where, e.g., users are running a TCP application from a mobile Wi-Fi station to an LTE UE, through a core. This is the domain of system-level simulators. For instance, the simulations reported in our previous papers that use SimuLTE (e.g., [18,27,28]), as well as in this one, could not be run using any of the above tools. With the exception of the Vienna simulator, these tools are single-purpose, and extensibility or interoperability are hardly addressed.

With system-level simulation tools, instead, extensibility and interoperability are major concerns, since these tools include different models, possibly contributed by different developers at different times. This is the case of LTESim [15] and the LENA LTE simulation library for ns3 [14]. A D2D module has been added to the latter recently [16], focused on network-unassisted D2D functionalities, namely out-of-coverage direct communications, direct discovery and synchronization. However, network-unassisted and network-controlled D2D communications are radically different, and there is little that one can use of either model to realize the other one. For instance, dynamic centralized scheduling and connection mode switching (which we describe in Section 5) are major issues in a network-controlled scenario, not so in an out-of-coverage one. The main characteristics of existing simulators are summarized in Table 1.

SimuLTE has been the subject of several conference papers so far [29,30,31,32,33]. The work in [29] describes the general outlook of an early version of the SimuLTE library. The modeling of inter-eNB X2 connections is described in [30], whereas the work in [31] shows how to make SimuLTE interoperable with the Veins vehicular network simulator [34]. The work in [32,33] introduces D2D communications and the configuration of scenarios involving them. Some book chapters [10,35] summarize the above papers, adding tutorial for configuration of the simulation scenarios. As a general note, the above literature has been written primarily for SimuLTE users, hence they describe SimuLTE capabilities, rather than discussing modeling choices and their implications. This paper fills the above gap, by taking a different perspective and describing in detail the modeling and implementation choices of the main features of SimuLTE, such as resource allocation, air transmission, and interference, most of which are as yet undocumented. Moreover, it shows how the initial modeling choices for the classical cellular communication allow an easy addition of D2D communications. Regarding the latter, modeling of P2MP D2D is entirely new, as is the modeling of mode switching in P2P D2D communications and frequency reuse in both P2P and P2MP D2D connections.

It is worth mentioning that SimuLTE is currently being used by a wide research community, that uses it to test its own research (such as schemes for mode selection [36], vehicular networks [37] or smart-grid monitoring [38]). The purpose of this paper is clearly not to discuss novel D2D-related algorithms, but to support (and possibly expand) the above community by presenting it with new models of D2D functionalities, and enabling it to assess the rationale and performance/accuracy implications of the key modeling choices.

## 4. Overview of the SimuLTE Architecture

As already stated, SimuLTE is a simulation library for system-level simulation of LTE networks. It is based on the OMNeT++ framework [12], which is built around the concept of module, a basic modeling unit which communicates via message exchanges and that can be hierarchically organized in compound modules. Each module is characterized by a structure, defined via Network Definition (NED) files, and a behavior, implemented via C++ classes. A general knowledge of the OMNeT++ framework is assumed henceforth. The interested reader can find more details on OMNeT++ in [39]. It goes without saying that SimuLTE modules can be instantiated in arbitrarily complex scenarios, involving any OMNeT++-compatible module: for instance, SimuLTE UEs can be vehicles moving about a floorplan according to Veins vehicular mobility model [34], communicating with fixed hosts provided by the INET library.

We now describe the architecture of SimuLTE. In doing so, we also describe and justify its core architectural choices—related to the modeling of a classical infrastructure-based cellular communication. These choices, in fact, guide the modeling of D2D functionalities, which is described in the next section.

### 4.1. General Architecture and Modeling of Protocol Layers

**Figure 3:**
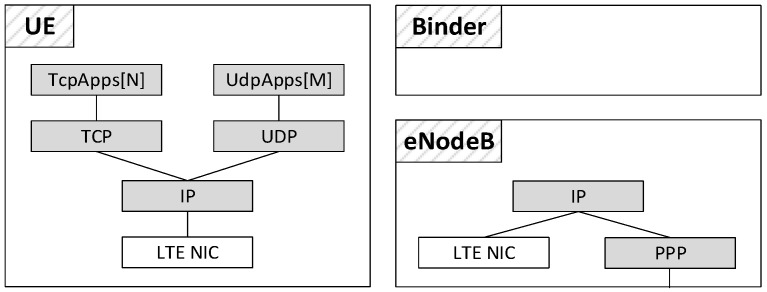
System overview of the radio-access network (grey: INET modules; white: SimuLTE modules).

The core SimuLTE module is the LTE Network Interface Card (NIC). LTE nodes, specifically UEs and eNBs, can be created by inserting into them the LTE NIC as a data link interface, and modules from INET for upper-layer protocols. The INET library is also used to implement entities outside the LTE scope, such as application servers, that are used as traffic generators/receivers and communicate with the application within the UEs. The NIC implementation allows one to develop nodes with multiple connectivity capabilities (e.g., LTE and/or Wi-Fi), and fully embodies the modularity paradigm on which the OMNeT++ framework is based. A high-level representation of the nodes involved in the radio-access network is given in Figure 3. Besides the LTE NIC, the UE module includes two vectors of application modules, which rely on either TCP or UDP transport protocols. The latter are built on top of the IP network-layer protocol. The eNB module, instead, has no transport or application levels. The IP layer at the eNB interconnects the radio interface (i.e., the LTE NIC) and the core network, accessible through the Point-to-point Protocol (PPP) interface. In addition to the models for the UE and the eNB, a module called Binder maintains data structures containing network-wide information and can be accessed via method calls by every node (both UEs and eNBs) in the network.

**Figure 4:**
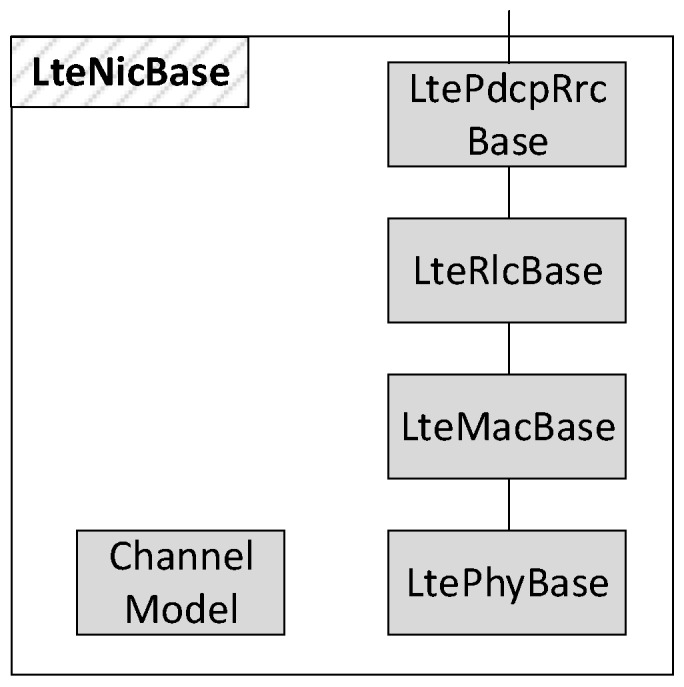
LTE NIC module architecture.

**Figure 5:**
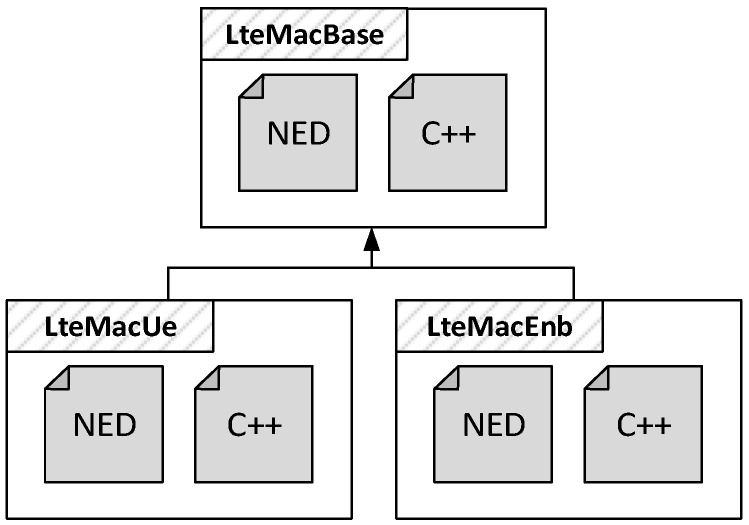
Example of inheritance of NED structure and C++ class.

The NIC modules in both the UE and eNB nodes implement the functionalities of the four layers of the LTE protocol stack, namely PDCP, RLC, MAC and PHY. For this reason, we modeled the LteNicBase module shown in Figure 4, which is composed of four submodules having a one-to-one correspondence with the LTE protocols. Another module, namely the ChannelModel, is introduced to simulate the effects of air transmissions, as we will explain in the next subsection. According to the OMNeT++ paradigm, each submodule is composed by a NED file and a C++ class defining, respectively, its structure and behavior. However, the same layer might perform different operations at the UE and at the eNB. For example, the MAC layer at the eNB carries out the resource scheduling, which is instead not performed by UEs. Thus, we extended the LteNicBase by LteNicUe and LteNicEnb modules. The latter include specialized versions of all the protocol layer’s submodules. With reference to Figure 5 and considering again the MAC layer as an example, we have the LteMacUe and LteMacEnb modules, both extending the LteMacBase NED structure and C++ class with node-specific functions, such as the resource scheduler on the eNB side.

### 4.2. Modeling Message Transmission and Interference

The main purpose of SimuLTE is to allow users to simulate the effects of LTE communications and resource allocation on the higher layers, e.g., to test resource allocation algorithms or to propose enhancements to protocol layers. An accurate modeling of OFDMA signal propagation, such as the one sometimes found in link-level simulators, is instead outside of its scope. This inspired specific choices regarding the modeling of the PHY and the air channel, which simplify the coding, improve the performance, and make the simulator more evolvable and maintainable.

The PHY of LTE is composed of several physical channels, which carry control and data information. For instance, downlink scheduling commands (i.e., which UE is the intended recipient of which set of RBs in a TTI) are sent on the Physical Downlink Control Channel (PDCCH), using a specific (and robust) modulation. The PDDCH is sent on the first three OFDMA symbols of a TTI. On the other hand, the MAC PDU for a given UE is sent on the Physical Downlink Shared Channel (PDSCH), using the modulation and coding chosen by the eNB. The PDSCH is mapped on the remaining symbols of the same TTI, which are transmitted later than the PDCCH’s. One could simulate transmission and reception of individual OFDMA symbols on both channels, apply some model of the path loss to individual symbols, and then come out with either correct reception or decoding errors. This is, in fact, the approach followed in [22], and it is time consuming from a performance standpoint.

**Figure 6:**
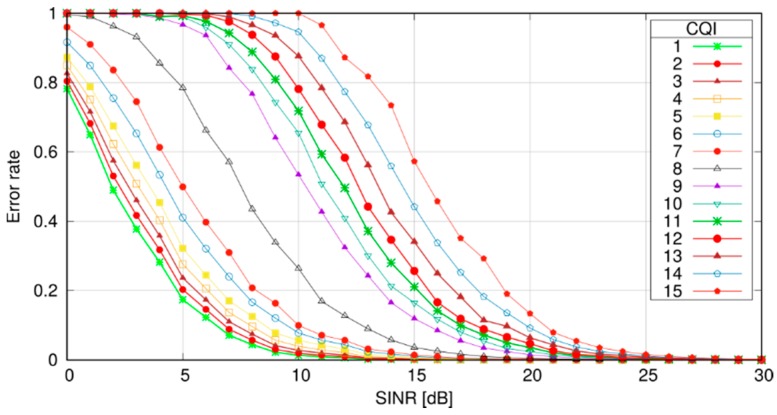
Example of BLER curves.

SimuLTE follows a different approach: the transmission of a MAC PDU (from the eNB to the UE in the DL, and vice versa for the UL) is modeled by having the transmitter’s PHY module send an OMNeT++ message to the receiver’s PHY. This message, sent through OMNeT’s sendDirect() method, contains a pointer to its MAC PDU. The recipient of such message then checks if the transmission was successful, by doing the following: for each RB *k* occupied by the transmission, the Signal to Interference and Noise Ratio (SINR) is computed according to the formula

$$SINR(k)=P_{RX}^{y}/N+\sum_{e\neq y}P_{RX}^{e}(k),$$

where the numerator is the power received from the transmitter and the denominator is the sum of the power received from every transmitter using RB *k*, plus noise. In both cases, the received powers are computed starting from the transmission power of the source, and applying the channel model for the channel between the recipient itself and the source. Once per-RB SINR have been computed, the recipient computes a per-RB probability of correct reception by using Block Error Rate (BLER) curves. BLER curves have the SINR as an abscissa and an error probability as an ordinate. Figure 6 reports sample BLER curves employed within SimuLTE, where the error probability decreases with the SINR for the same CQI value. More efficient modulations (i.e., CQIs) require larger SINRs to allow correct decoding. BLER curves are available as outputs of link-level simulations (see, e.g., [40]), but can also be replaced by the user if needed. The per-RB error probabilities are then combined to obtain a per-transmission error probability, assuming independence between RBs and using standard probability manipulations. The correct reception is then assessed by extracting a random value from a standard uniform distribution and checking whether it is below or above the per-transmission error probability.

According to the above modeling, the recipient of the OMNeT++ message receives *only* the message intended for it, whereas it does not receive messages transmitted to other destinations. This means that the information about interfering transmissions must be obtained through different means. In the DL, this is fairly straightforward: each eNB generates a map of the allocated RBs, as part of its resource allocation process, as explained in the next subsection. Therefore, the list of which eNB uses which RB can be constructed by simply querying the eNB’s MAC layers. Since a transmission occurring at TTI *t* is decoded at TTI *t +* 1, each eNB must keep track of the allocations at the previous TTI, which is done by flipping between an actual and a past allocation maps on each TTI. For the UL transmissions, however, the situation is slightly more complex. The eNBs do have a map of which UE was granted which RBs on each TTI, but UEs may use a grant only partially or not at all, hence relying on the eNB allocation maps would sometimes create inconsistencies. Our choice is to maintain a global UL transmission map (UTM) data structure (stored within the Binder) that keeps track of which UEs were using which RBs at both the current and the previous TTIs. When a UE’s PHY layer transmits the message, it registers which RBs it is using in the Binder’s UTM. The receiving eNB, then, computes the set of interferers on each RB by scanning the UTM. Note that the number of UEs using a given RB is at most one per cell (unless frequency reuse is in place, which happens with D2D transmissions—see Section 5.2), hence interference computation is generally fast.

The above mechanism allows a correct accounting of the effects of interference, as well as arbitrarily complex channel models, without the need to simulate symbol transmission at the PHY layer. SimuLTE defines the ChannelModel as an interface, i.e., a C++ abstract class with pure virtual functions only, and also provides an implementation of a realistic model, which accounts for pathloss, fading and shadowing.

There are several benefits in using the above approach: the first one is simplicity of implementation, which is self-evident. The second is scalability: in a simulation with many UEs *N*, every UE should listen to the PDCCH every TTI, most often to discover that there is no information for it on the PDSCH. This requires O(*N*) operations per TTI. Using the above approach, only the (considerably fewer) UEs that are recipients of an ongoing transmission, let them be $N_{r}$, receive a message through a sendDirect(). These are at most as many as the RBs in a subframe, *M*, and often considerably fewer, since a MAC PDU may occupy more than one RB. Since $N_{r}\leq M<<N$, this makes the simulator more scalable. For instance, in the simulations in [27], with UEs receiving VoIP, $N\approx 31\cdot N_{r}$. A third, non-negligible benefit is evolvability and upgradability: more physical transmission schemes at the PHY of LTE have been added throughout the releases. Adding new physical transmission schemes (e.g., a new higher-order modulation) in SimuLTE only requires adding the related BLER curves, and possibly specializing the ChannelModel accordingly. As we shown below, the above modeling is in fact what made adding D2D to SimuLTE a relatively simple effort.

Of course, the above modeling approach has drawbacks as well: for instance, it makes SimuLTE unsuitable to evaluate physical-layer details and functions, such as transmissions schemes or receiver algorithms, which have to be abstracted to fit our model. As already observed, this is in fact the domain of link-level simulators (e.g., [22]).

### 4.3. Resource Allocation at the eNB

**Figure 7:**
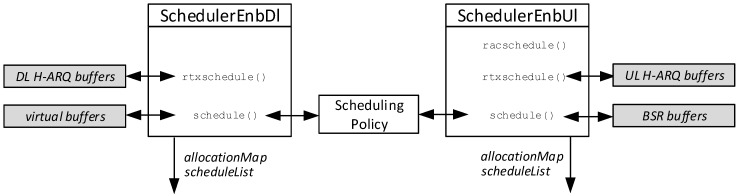
Scheduling operations.

Resource allocation is done at the MAC layer of the eNB. Our model of the latter contains two classes, namely LteSchedulerEnbDl and LteSchedulerEnbUl, which perform the scheduling of DL and UL frequency resources to backlogged UEs on each TTI and are represented in Figure 7.

**Figure 8:**
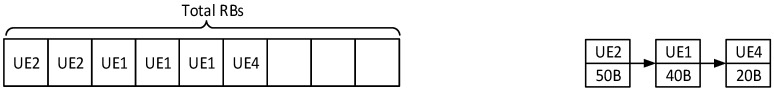
Allocation map (**left**); and schedule list (**right**).

Considering the DL direction, the LteSchedulerEnbDl function first checks whether some MAC PDUs in the H-ARQ buffers are awaiting retransmission through the rtxschedule() function and allocates resources to them. Then, the schedule() function allocates resources for new data transmissions. Recall that the data that will form MAC PDUs are fetched from the eNB’s RLC buffer. Thus, the LteSchedulerEnbDl should query the RLC module whenever it needs to know the amount of buffered data, i.e., at least once per TTI and for all the UEs under the control of the eNB. This would require O(N) OMNeT++ messages per TTI to be exchanged between the MAC and RLC modules. To abate this overhead, we endowed the eNB MAC module with virtual RLC buffers (VRBs), storing the length of each UE’s RLC buffers directly in the MAC. VRBs are updated only when a new RLC SDU is received from the PDCP, in which case the RLC module send one OMNeT++ message to the MAC indicating the length of that SDU (and this is unavoidable), and upon transmission of a MAC PDU, by subtracting the relevant amount of dequeued bytes at the RLC. All the lookups of the UE’s RLC buffers required by the LteSchedulerEnbDl on each TTI are instead done on the VRBs, which saves a considerable overhead. The LteSchedulerEnbDl builds a set of active UEs, i.e., those with a non-empty RLC buffer, and runs a scheduling policy that sorts them according to a specified policy. Note that the scheduling policy is a separate plugin entity: this allows SimuLTE to implement several policies that can be used interchangeably, e.g., MaxC/I or Proportional Fair. Then, the scheduler scans the sorted list and allocates a number of RBs to the UEs depending on its CQI and the length of its VRB. The operation terminates when the list is empty or there are no more available RBs. At the end, the scheduler obtains an allocation map (described previously) and a schedule list, shown in Figure 8. The latter indicates how many bytes are served for each UE and is used to request the RLC module to send down one RLC PDU of the given size for each UE. The MAC module forms the TB by adding its header to such RLC PDU and sends it to the PHY module.

UL scheduling mirrors the above checklist, with some peculiar differences. The first function called by the LteSchedulerEnbUl is the racschedule(), which allocates one RB to UEs that performed a successful RAC request during the last TTI, which the latter normally use to transmit their BSR (see Figure 2). Moreover, the eNB maintains BSR buffers, which store the estimated backlog of UEs, and are updated on reception of new BSRs from the UEs and decreased on reception of traffic from UEs. The alert reader will note that BSR buffers play exactly the same role as VRBs in the DL, i.e., that of enabling the eNB MAC to estimate the backlog for a UE. In fact, we exploited this to make scheduling policies independent of the scheduling direction: the same scheduling policy can work seamlessly for both the UL and the DL.

Finally, we observe that, in the UL, the scheduling operation consists in sending a scheduling grant to each UE in the list. The latter is a control packet indicating how many bytes it can transmit, using which RBs.

## 5. Modeling Device-to-Device Communications in SimuLTE

SimuLTE implements both P2P and P2MP D2D communications. In what follows, we show the main modeling choices regarding both. These concern: modifications to the protocol stack to incorporate the new functions required by D2D; modifications to resource allocation at the eNB, to account for frequency reuse and mode selection; and modeling air transmission, i.e., identifying the set of possible receivers for a transmission and modeling its logical and physical effects.

### 5.1. Modifying Protocol Layers to Accommodate D2D Data Processing

**Figure 9:**
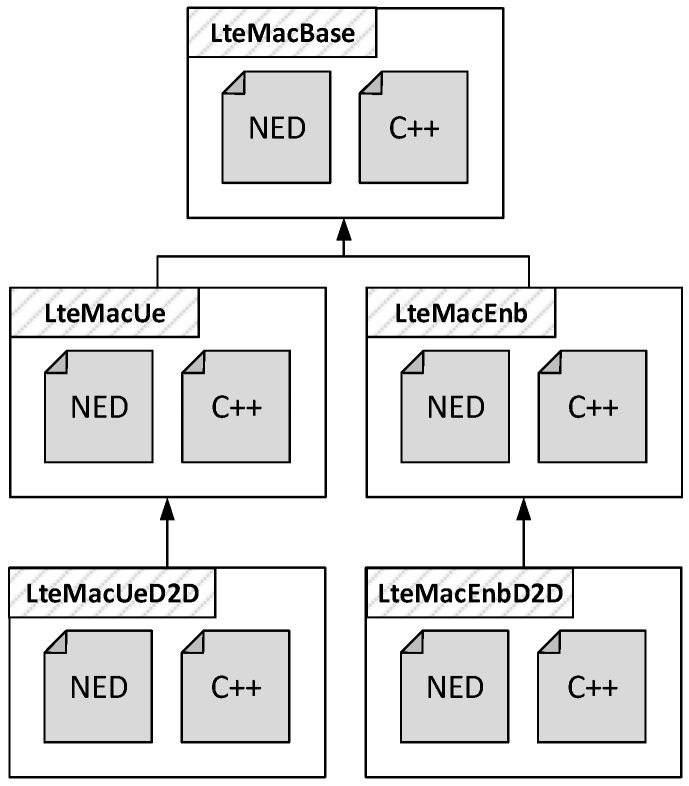
Example of inheritance of NED structure and C++ class for D2D-specific modules.

As discussed in Section 2, D2D communications traverse all the layers of the LTE protocol stack, top-down in transmission and bottom-up in reception. This allowed us to reuse most of the modeling done for traditional cellular transmissions exploiting inheritance and the modularity of OMNeT++. Thus, for each protocol layer requiring additional D2D-specific operations, we modeled extended versions of the relative submodules, as shown in Figure 9 for the MAC layer. In this case, LteMacUeD2D adds, for example, support for H-ARQ retransmissions for D2D flows and other functionalities described in the following, whereas LteMacEnbD2D provides handling of D2D connections during the resource allocation.

We first describe the few modifications required to allow a correct traversal of the protocol stack for transmission/reception of D2D data, and then discuss the more complex issue of mode switching (MS), i.e., what happens when a P2P D2D flow is switched from the SL to the infrastructure path or vice versa.

**Figure 10:**
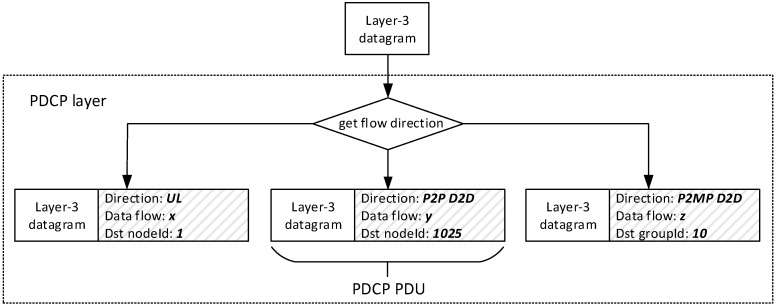
Processing of upper-layer packets at the PDCP layer.

With reference to Figure 10, IP datagrams arriving at the PDCP of the sender UE are associated to a data flow identified by a 4-tuple composed by source/destination IP address and source/destination port. Moreover, they are assigned a direction, which can be UL or the newly added P2P and P2MP D2D. This way, data having the same 4-tuple, but different flow directions, will belong to different, independent data flows, allowing lower layers to treat them accordingly. In fact, the flow direction for the same source–destination pair can be changed dynamically because of mode switching*,* discussed later. Regarding P2MP D2D communications, our implementation maps multicast IP datagrams (i.e., those with a multicast IP destination address) to the P2MP D2D flow direction. The IP datagram is also assigned a multicast group ID by querying the Binder. Finally, the PDCP layer attaches to the resulting PDCP PDU the node ID (or the group ID) of the intended receiver(s), which will be used by lower layers.

The RLC layer has no specific functionalities connected to the top-down traversal of D2D data. The latter are stored in RLC transmission buffers just like UL data. However, the RLC plays a pivotal role during MS, as we discuss below.

As shown in Section 2, the MAC layer of the UE must notify the eNB when new data are available for transmission, by sending it a BSR. We have added to the BSR a specific field to distinguish among UL, P2P D2D, and P2MP D2D BSRs. The eNB will use this information to issue transmission grants for the correct direction. Other than this, the operation at the MAC layer of a D2D connection are the same as for UL transmissions, with the only exception that the functions and data structures to handle H-ARQ processes are disabled with P2MP D2D transmissions.

**Figure 11:**
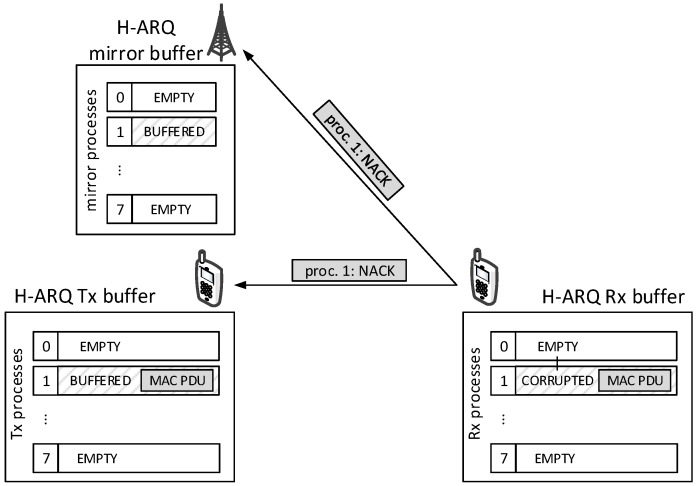
Example of H-ARQ buffer mirroring.

Let us discuss now what happens at the receiver side of a D2D connection, i.e., the bottom-up traversal of the LTE protocol stack for D2D data. The main difference lies within the MAC layer, whereas the RLC and PDCP layers are unaffected (the same operations required for non-D2D data are performed with D2D data as well). As far as P2MP D2D data are concerned, MAC-layer processing is a simplified version of the one for non-D2D data, the simplification being that no H-ARQ feedback is generated. In our modeling, P2P D2D communications are instead acknowledged at the MAC layer, i.e., the receiver sends H-ARQ feedbacks (ACKs/NACKs) upon reception of MAC PDUs. While there is no standard prescription on how to do this, our understanding is that ACKs/NACKs are needed at both the transmitting endpoint (another UE) and the scheduling entity (the eNB), since both need to take action according to the feedback: the transmitting endpoint has to either free or keep busy the H-ARQ process involved in the transmission, and the scheduling entity has to schedule retransmissions of a busy H-ARQ process at the transmitter when the receiver sends a NACK. Therefore, coherently with [18], we assume that the eNB overhears the H-ARQ feedback sent by the receiving UE to the transmitter UE. This assumption is sound, since H-ARQ control channels in LTE use a fixed, robust modulation, and are power-controlled to reach the eNB. With reference to Figure 11, the above assumption is realized by sending the ACK/NACK through a sendDirect() to both the transmitter and the eNB, including a counter indexing the process that the feedback refers to. The transmitter updates its H-ARQ process the same way it would in an UL transmission (where it would receive the same feedback from the eNB itself). The eNB’s MAC layer uses the feedback to update a data structure that mirrors the status of each H-ARQ Tx buffer on the transmitter, consisting of a vector of eight elements (i.e., one per H-ARQ process). Note that P2P D2D data transmissions are not overheard by the eNB, hence the contents of the H-ARQ Tx buffers are not stored at the eNB.

In P2P D2D communications between two UEs, several factors, such as mobility, interference and network conditions, might affect the performance and, in some cases, trigger a mode switch of the communication, i.e., changing the communication mode to the infrastructure path or vice versa. For example, when the two endpoints move far from each other and get out of the respective hearing range, switching to the infrastructure mode is the only way to keep the connection alive. Conversely, two endpoints that were too far for D2D communications may become near enough, hence switching them to the SL could improve their performance. MS is also advisable when one endpoint of a D2D communication initiates handover: maintaining a direct communication between two UEs located in different cells would require that the corresponding eNBs coordinate their resource allocation in a tight lockstep [18], which is computationally costly. Direct communication might then resume if and when both UEs are again under the control of the same eNB.

**Figure 12:**
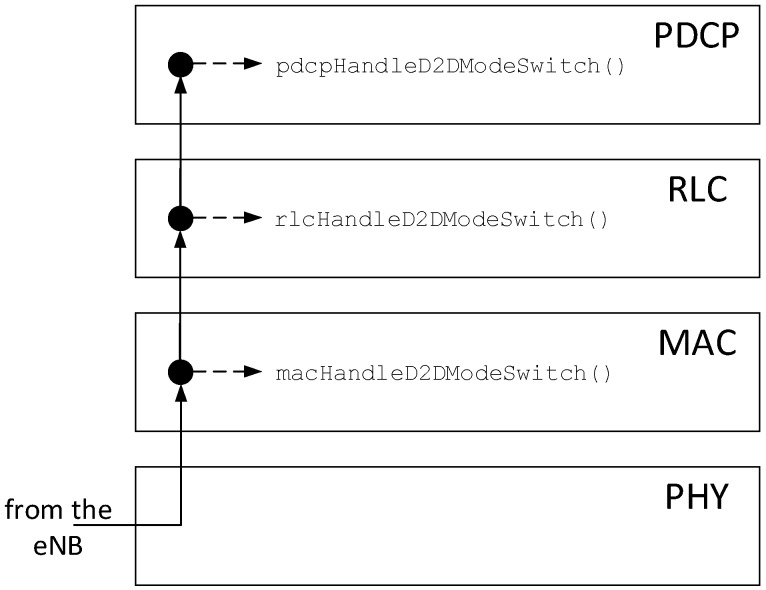
Management of the mode switching.

MS decisions are made by the eNB, which informs both UEs about the path that they should use thereafter. However, a MS requires actions to be taken at every layer of the LTE protocol stack at the UEs’ side (e.g., clearing buffers at MAC and RLC layers). In SimuLTE, we implemented the MS notification as an OMNeT++ message generated by the eNB’s MAC layer and sent (through its PHY layer) to both the endpoints of the P2P D2D communication. This message reaches the destinations’ PHY layer in one TTI and it is always decoded with no errors. With reference to Figure 12, the message traverses all the modules implementing the layers of the LTE protocol stack. Every layer provides a handler that is invoked upon reception of the notification from the lower layer, accomplishes the required MS operations for that layer, then it forwards the message to the upper layer. This implementation allows one to write its own handlers to implement different MS schemes. Examples of MS decisions at the different layers are the following:

- At the MAC layer of the transmitter, how to manage busy H-ARQ processes, e.g., those waiting for an acknowledgement or a grant for retransmitting a MAC PDU. One choice may be to interrupt them, hence dropping MAC PDUs awaiting retransmission. Alternatively, one could allow H-ARQ processes to terminate their cycle along the “old” path before switching to the “new” one.
- At the RLC layer, whether to flush the RLC buffer (which contains data ciphered with the wrong session key, see [18]) and switch mode instantly, or to defer switching until the RLC buffer has been drained by the MAC.
- At the PDCP layer, whether to buffer SDUs in anticipation of a possible future MS operation, and whether to relay them to the PDCP entity that manages the new direction upon MS (see again [18]).

### 5.2. Modeling Resource Allocation

D2D transmissions require resource allocation to be modified. As explained in Section 2, D2D transmissions are allocated on the UL subframe, hence D2D and UL transmissions share the RBs of the UL subframe. Our solution is to enforce no static segregation between RBs “dedicated” to one or the other, since this leads to inefficiencies. The only case when static segregation is in place is when ARS allocation is enforced (typically, for P2MP communications): in this case, in fact, resources are allocated statically according to the standard, and accessed autonomously (and with contention) by the UEs. In all the other cases where resources are scheduled, all UEs can be scheduled anywhere anytime.

Given that D2D transmissions share RBs with the UL ones, we enhance the LteSchedulerEnbUl class at the eNB’s MAC to include both P2P and P2MP D2D connections in the scheduling process. This only requires that the eNB MAC maintains BSR buffers for D2D connections as well: these BSR buffers are updated on reception of P2P D2D and P2MP D2D BSRs from the sender UEs of a D2D connection. Apart from that, the UL scheduling operations described in Section 4 are unmodified. The resulting schedule list is composed of UL, P2P D2D and P2MP D2D connections, arbitrarily interleaved according to the employed scheduling policy. When scanning the schedule list to send transmission grants to the UE, the eNB MAC module takes care to include in the control packet the direction the granted RBs refers to.

**Figure 13:**
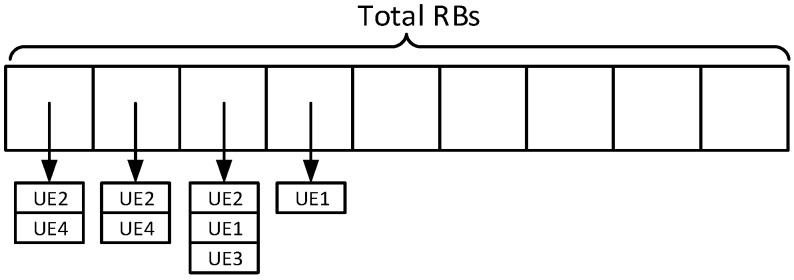
Allocation map with frequency reuse.

As mentioned above, scheduled D2D transmissions can exploit spatial reuse, i.e., allocate the same RB to more than one connection (under controlled circumstances), to reduce the frame occupancy. To allow for this, the LteSchedulerEnbUl class is adapted to produce an allocation map that allows it to store more than one transmitting UE in one RB. Figure 13 exemplifies such modified data structure, where each entry (i.e., each RB) contains a set of UEs.

**Figure 14:**
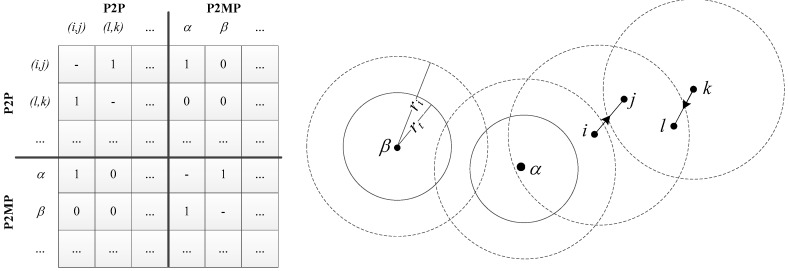
Example of conflict matrix (**left**); and a node disposition which produces it (**right**).

Our support for D2D-aware resource allocation algorithms in SimuLTE allows one to enforce frequency reuse among D2D connections (both P2P and P2MP), but prevents two UL connections in the same cell to share resources. To keep track of reuse, we make available a data structure that computes potential conflicting transmissions based on a topological approach. In the latter, we assume that the position of the UEs in the floorplan is known. The above assumption is being widely used in the literature (see, e.g., [41,42]), and becomes more credible with the spread of location-based services and Multi-access Edge Computing. The eNB MAC module can get the position of each UE by querying the Binder, and uses it to build a Conflict Graph (CG), i.e., a symmetric graph that states which pairs of transmitters conflict with each other, hence should not be activated simultaneously. The CG can be represented in matrix form. With reference to Figure 14, the topmost diagonal block of the conflict matrix stores inter-P2P conflicts, whereas the bottom diagonal block stores inter-P2MP conflicts. Both blocks are symmetric. The non-diagonal blocks hold P2P-P2MP conflicts. The heading of each row/column is the name of the related flow, and we use a couple of lowercase letters to indicate the transmitter and receiver for P2P flows and a single Greek letter to indicate the transmitter of a P2MP flow. Each transmitter has two radiuses: a transmission radius $r_{t}$, which corresponds to the maximum distance at which a receiver has a “high” probability of decoding, and an interference radius $r_{i}$, $r_{i}>r_{t}$, corresponding to the maximum distance where its interference is considered to be detrimental to other receivers. These radiuses can be set based on the transmission power and the pathloss model. Assuming—for ease of exposition only—that the values of $r_{t}$ and $r_{i}$ are the same for all transmitters (we leave to the interested reader the simple, yet tedious generalization to the case of per-flow transmission and interference radiuses), and denoting with d(i,j) the Euclidean distance between UEs *i* and *j*, the conflict conditions are the following (see the example in Figure 14):

(1) Inter-P2P: two P2P flows (*i*,*j*) and *(k*,*l*) are conflicting if $\min\left\{d\left(i,l\right),d\left(k,j\right)\right\}\leq r_{i}$
(2) Inter-P2MP: two P2MP transmitters α and β are conflicting if $d\left(\alpha ,\beta \right)\leq r_{t}+r_{i}$
(3) P2P with P2MP (and vice versa): flow (*i*,*j*) and transmitter α are conflicting if $d\left(i,\alpha \right)\leq r_{t}+r_{i}$

Note that the above conflict conditions may be more restricting than necessary. For instance, in Condition (3), the hidden hypothesis is that there actually *is* at least one P2MP receiver UE *x* such that $d\left(\alpha ,x\right)\leq r_{t}$ and $d\left(i,x\right)\leq r_{i}$, which may or may not be the case. Testing for the location of P2MP receivers, however, is computationally complex (it may require up to O(N) operations per conflict), whereas each of the above three conditions can be tested in constant time.

The above data structure can be used to devise reuse-aware schedulers, such as the one in [28], that prevent a priori resource sharing among some couples of transmitters. We remark that physical interference on D2D transmissions is always computed according to a SINR model a posteriori, as explained in Section 5.3, hence the cumulative effects of small interferers would be felt by transmissions, though not anticipated by inspecting the above data structure. The main drawback of this approach is that algorithms allocating resources based on the conflict model cannot foresee the cumulative effect of small, faraway interferers [43]. We evaluate this phenomenon in Section 6.2.

Finally, we want users to be able to choose, at configuration time, which type(s) of D2D connections should exploit frequency reuse. For instance, a user may want to limit frequency reuse to P2P D2D connections, scheduling P2MP ones on exclusive resources. To make this efficient, we modeled SimuLTE scheduling so that connections which are not in the conflict graph are to be scheduled on mutually exclusive resources by default. Therefore, if a user configures a simulation where frequency reuse occurs only among P2P D2D connections, then only the topmost diagonal block of the conflict matrix will be instantiated and used, with reduced scheduling complexity.

Besides managing resource allocation, the eNB has to perform mode selection for P2P D2D connections, i.e., decide which of them should be allocated on the SL and which on the UL/DL infrastructure path. Several papers advocate joint mode selection and resource allocation (e.g., [44,45]), whereby the eNB does both at the same time. We disagree with the above approach, since mode-selection decisions (especially on a cell-wide basis) may take a considerable amount of time, and the ensuing MS operations may induce losses [18], hence they cannot be too frequent (certainly not every TTI). On the other hand, resource allocation must be done on a per-TTI basis to capitalize on the knowledge of fresh channel conditions and recent backlog. For this reason, in SimuLTE, mode selection is autonomous, and can be configured to run at the eNB at the desired frequency (we suggest once every 100–1000 TTIs, a timeframe comparable with appreciable changes in the relative distance of UEs in motion, hence their channel). We support mode selection schemes that can take into account both local and global (i.e., cell-level) metrics. For example, even if the SL between two D2D endpoints is experiencing poor channel conditions, switching it to the infrastructure mode might still be unadvisable, e.g., because the DL subframe is overloaded.

Mode selection algorithms can be written by extending the D2DModeSelectionBase module residing at the MAC layer of the eNB. Such module periodically runs a doModeSelection() function that produces the communication modes to be used in the next period for all the ongoing D2D peerings. Custom mode selection policies can be implemented by inheriting the structure of the D2DModeSelectionBase module and overriding the doModeSelection() method. An example of a mode selection algorithm can be found in [28].

### 5.3. Modeling Air Transmissions of D2D MAC PDUs

The modeling paradigm for air transmissions in SimuLTE, exploiting direct OMNeT++ messages, on the one hand, and SINR computation at the receiver, on the other, naturally lends itself to account for D2D transmissions. The first issue to be solved is endpoint identification, i.e., to keep track of which pairs (groups) of UEs are eligible as endpoints of a D2D communication at any time.

**Figure 15:**
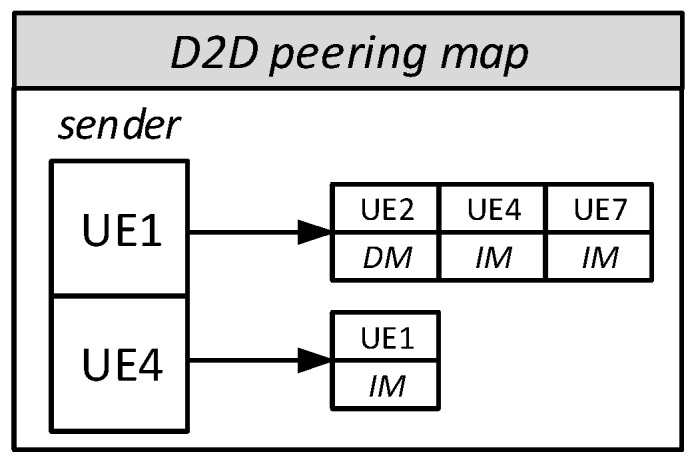
D2D peering map (DM: direct mode, IM: infrastructure mode).

For P2P D2D, SimuLTE maintains a peering map within the Binder, as shown in Figure 15. For each D2D-capable sender *s*, the map contains the set of possible destinations for which a D2D peering session might be established at some point, i.e., all the D2D-capable receivers to which *s* has already sent a message. This last condition keeps the map compact, since considerably fewer P2P flows are simulated than the cross-product of all the D2D-capable UEs. When a UE receives an IP datagram to be sent to a new destination, it checks through the Binder if the latter is a D2D-capable LTE device, in which case it adds the new related entry to the map. Each eNB performs mode-selection decisions on those flows in the peering map having both endpoints under its control (P2P D2D flows across cell borders are not supported in SimuLTE, and we believe that they are generally unadvisable, as discussed in [18]), and stores the result directly in the peering map. By default, the communication mode is set to infrastructure, and is modified through MS operations. Moreover, when two endpoints in the peering map are under the same eNB, CQI reporting on the SL is also enabled. When a new IP datagram arrives from the upper layers, the UE’s PDCP checks the peering map to associate the packet to either the UL or the D2D direction. In the latter case, the node ID of the peering UE selected as destination ID, which represents the argument of the single sendDirect() invocation used to send a message.

For P2MP D2D, the problem is to identify the interested receivers of a multicast transmission. In SimuLTE, we use the concept of multicast groups, each identified by an ID: at the reception of an IP datagram from the upper layers whose destination address is a multicast one (i.e., comprised within the range 224.0.0.0–239.255.255.255), the UE’s PDCP layer considers the last eight bits of such address as the destination ID. Coherently, the packet is associated to the P2MP D2D direction. The sender UE then creates a copy of the MAC PDU for every UE in the multicast group (identified by the destination ID) and invokes the sendDirect() accordingly. From a physical standpoint, each receiver of a sendDirect() (originating from either a P2P or a P2MP transmission) computes the SINR as shown in Section 4.2, irrespective of who the sender is (an UE PHY module instead of an eNB’s). All it takes is to add D2D transmitters to the UTM, so that they contribute to the general interference in the UL spectrum (for both SL and—less critically—UL transmissions).

For P2MP transmissions, an optimization has been implemented to reduce the number of sendDirect() invocations and ensuing SINR computations at the receivers. Since D2D transmissions are low-power, there exists a physical transmission horizon for each sender, beyond which successful decoding would be vanishingly unlikely in any case, because the received power would be too small. Such horizon can be computed based on the transmission power. All the destinations beyond the horizon line could then be eliminated from the target list of a message, with a relatively small loss in modeling accuracy. Below, we show how this optimization allows a remarkable speedup in some scenarios (e.g., vehicular communications with macro cells), where membership of the same multicast group is geographically diffuse, hence there is a high probability that the distance between two members is considerably larger than a transmission range.

Another issue to be considered at the receiving side is that a UE might be the intended target of multiple simultaneous and overlapping D2D transmissions. This may happen, for instance, when ARS allocation is in place and two or more members of the same multicast group attempt to use the same RBs simultaneously. In this case, the receiver will attempt to decode only the MAC PDU with the highest received power, according to the capture effect. All the other simultaneous transmissions are instead treated as interference. SimuLTE models the capture effect by postponing decoding to the end of the TTI: during a TTI, UEs just store all the messages received during a TTI, along with their received power, without performing decoding operations. At the end of the TTI, the MAC PDU with the highest received power is selected and passed as an argument to the error() function, whereas the other ones are simply discarded.

## 6. Performance Evaluation

This section includes two contributions: first a profiling of SimuLTE execution time, varying the number of UEs and the packet rates, also assessing the impact of D2D communications; and, second, an analysis of D2D-enabled scenarios, whose purpose is to show the impact of some of the factors that we described in Section 5 (namely, the interference radius, the mode-switching policy and the transmission horizon) on the observable performance.

### 6.1. Profiling

The purpose of this section is to assess the computational overhead of D2D communications in SimuLTE. More specifically, we want to show that adding D2D communications increases the simulation time only marginally with respect to classical infrastructure-based communications.

We profile the execution time of SimuLTE, analyzing how the latter is influenced by the number of UEs and the system packet rate. Moreover, we verify the impact of the D2D implementation on the execution time, by executing three variants of the same scenario, where several UEs send each other packets using, respectively, infrastructure communication (henceforth referred to as Infra), or D2D one-to-one communications, with and without dynamic transmission format selection (referred to as D2D and D2D-Fixed, respectively). The scenario consists of a single cell, where D2D endpoints are close to each other and to the eNB, so that all CQIs on the UL, DL, and SL are equal to 15. Each D2D pair supports a unidirectional flow, with a constant inter-packet time and packets of fixed size throughout the whole simulation. eNB scheduling is done without frequency reuse. The above scenario is run for 100 s of simulated time, five times in independent conditions. We perform our experiments on a Kubuntu 16.04 machine equipped with an Intel(R) Core(TM) i7 CPU at 3.60 GHz, with 16 GB of RAM. Execution times are measured using the *time* Unix command on batch and command-line only simulations, i.e., without any graphical user interface.

**Figure 16:**
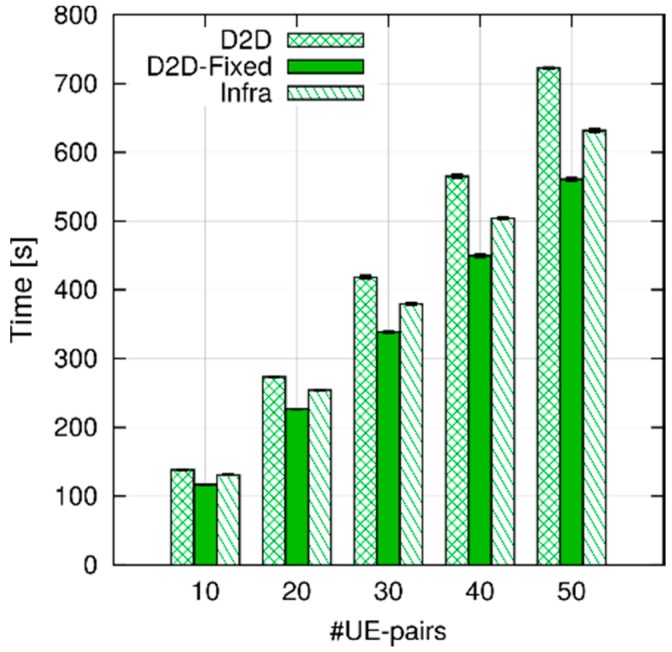
Execution time for an increasing number of UEs, obtained with a per-UE inter-packet-time of 0.1 s.

**Figure 17:**
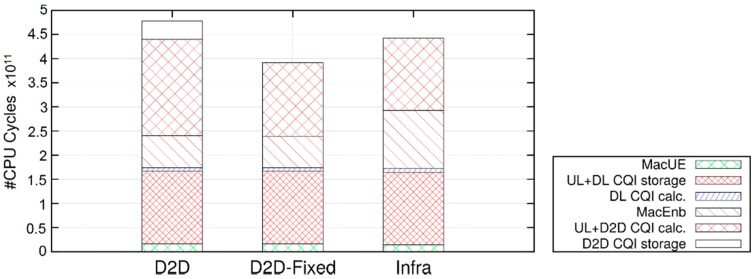
Breakdown of execution times with 10 UE pairs, 0.1 s inter-packet time (leftmost bars in Figure 16).

In Figure 16, we compare the execution time in the three configurations, varying the number of UE pairs, while maintaining the inter-packet time at 0.1 s. First, we can see that the execution time grows almost linearly with the number of UE-pairs. Second, the Infra configurations are faster than the D2D one, but slower than the D2D-fixed. On the one hand, in the D2D configuration CQIs for both the UL and the SL must be computed, which requires twice as many channel computations as the Infra and D2D-Fixed configurations. On the other hand, infrastructure-based communications require two MAC-layer transmission (i.e., in the UL and the DL legs) to get a packet to destination, whereas D2D ones take a single hop, which reduces the number of MAC-layer computations. The above explanation is confirmed by the breakdown of the execution times, which can be obtained by running a simulation through *valgrind* (http://valgrind.org) (measurements are obtained using *callgrind*, one of valgrind’s tools, which counts the number of CPU cycles spent within each function) and observing the time spent performing each high-level function. One such breakdown is shown in Figure 17. While absolute times are not comparable to those of Figure 16, since using valgrind slows down the simulation considerably, the figure shows that the Infra configuration spends more CPU cycles than the other two running the MAC at the eNB (because of the two-hop communication), whereas the *D2D* configurations spends more time computing and storing SL CQIs.

**Figure 18:**
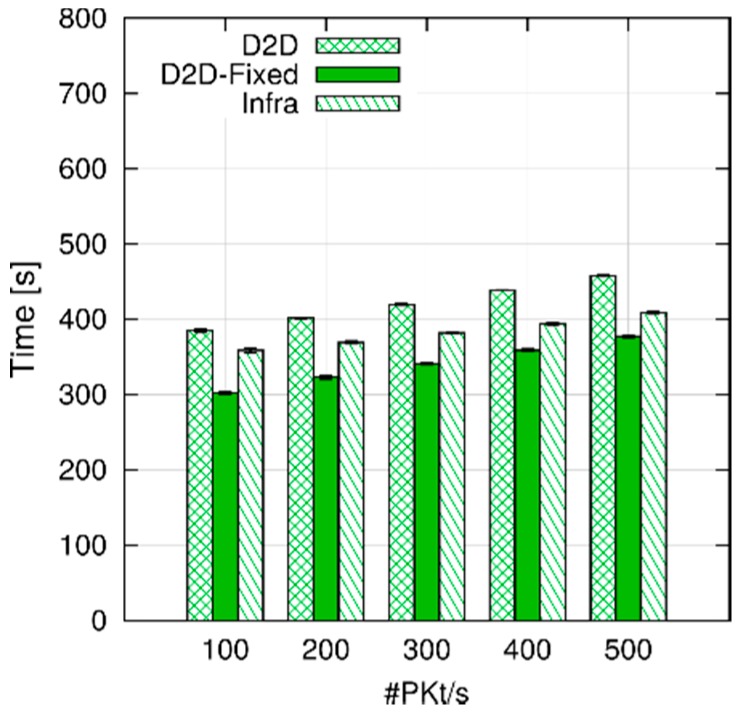
Execution time for an increasing system packet rate, obtained with a fixed number of 30 UEs.

In the previous experiment, we used a fixed inter-packet time of 0.1 s. Therefore, the overall number of packets per second in the simulation increased with the number of UEs. In Figure 18, we show the results obtained fixing the number of UE pairs to 30, and reducing the inter-packet from 0.3 to 0.06 s, thus obtaining a cell-wise packet rate from 100 to 500 pps (In the last configuration, ~20% of the LTE system spectrum is allocated on average, corresponding to a medium load). As we can see, this leads to a small increase in the execution time, and the same relationships among the three scenarios are still observable. The influence on the execution time of the number of UE pairs and system packet rate in the D2D scenario can be formally assessed through 2*^k^r* factorial analysis, using the parameter ranges in Table 2. The results in Table 3 show that about 98% of the variation in the execution time is due to the number of UE pairs, and that the remaining 2% is due to the packet rate. The reason behind this large imbalance (confirmed by Figure 17) is that CQI computation and storage, which is done per UE, is performed quite frequently (every 5 ms), and it involves a relatively large amount of computations, whereas MAC-layer transmissions occur comparatively less often.

Observing the above results, we can summarize that introducing D2D communications does not increase the processing overhead of packets flowing within the LTE protocol stack. On the other hand, the execution time is affected by the need of computing and storing additional channel information for D2D links.

### 6.2. Evaluation of D2D-Enabled Simulation Scenarios

Adding D2D communications to SimuLTE allows one to analyze how different parameters and algorithmic choices affect the performance at different layers of the protocol stack. We discuss three exemplary scenarios: In Scenario 1, we show that selecting different values of the interference radius affects the allocation of resources and the resulting SINR when frequency reuse among D2D transmissions is enforced. In Scenario 2, we highlight the interplay between mode switching of D2D communications and upper protocol layers, i.e., transport and application layers. More in detail, we observe that different mode-switching policies within the LTE protocol stack imply different performance of applications running on top of UDP and TCP protocols. In Scenario 3, we show that a careful selection of the transmission horizon for P2MP D2D communications leads to significant speedup of the execution time, without affecting the accuracy of the results.

**Scenario 1—Selection of the Interference Radius**

**Figure 19:**
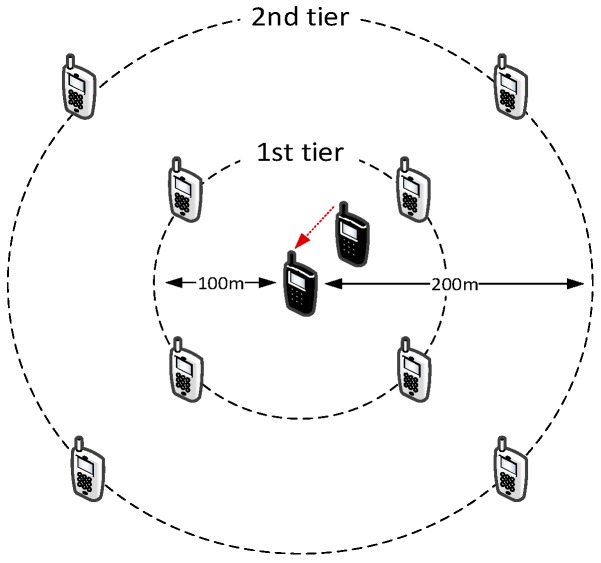
Simulation scenario for analysis of frequency reuse.

**Figure 20:**
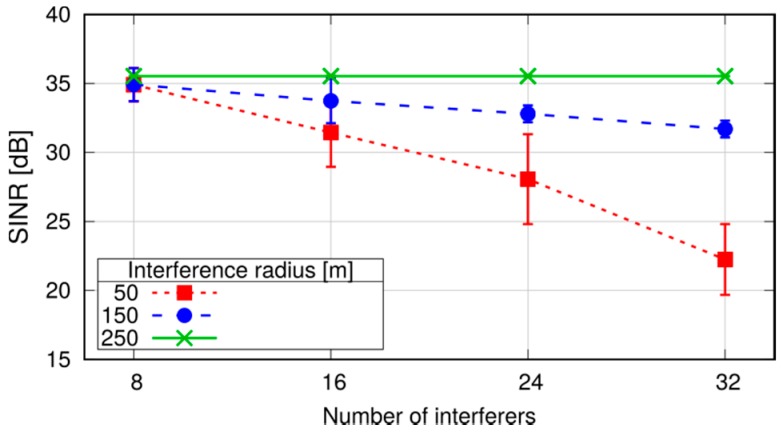
SINR perceived with different interference radiuses.

We now evaluate the effects of using a Conflict Graph (CG) approach to make decisions about frequency reuse in D2D resource allocation. Figure 19 shows the simulation scenario, where we observe the SINR perceived by the receiver of one D2D connection, located 50 m from its transmitter. The observed D2D connection (the black UEs in the figure) is surrounded by two tiers of interferers, deployed at 100 and 200 m, respectively, from the receiving UE. For ease of reading, only the transmitting endpoints of the interfering connections are displayed in the figure. Each transmitter sends a 100-byte packet every 20 ms. We vary the number of interferers from 8 to 32. The main simulation parameters are reported in Table 4, column *Scenario 1*. To highlight the effects of the CG, we disable channel fading, so that the received power depends only on the distance to the transmitter. We vary the interference radius $r_{i}$ used to build the CG, setting it at 50, 150 and 250 m: with $r_{i}=50$ m, the CG is empty and the tagged D2D connection can share resources with all the other transmitters; with $r_{i}=150$ m, the first tier of interferers is marked as conflicting; and, with $r_{i}=250$ m, both tiers are marked as conflicting, hence the tagged D2D connection transmits in mutually exclusive RBs. Figure 20 shows that setting $r_{i}$ to 150 m allows a relatively large number of simultaneous transmissions with only a limited decrease in the SINR. Figure 20 also confirms that, although the CG allows multiple transmitter whose distance is larger than $r_{i}$ to share the same RBs, the cumulative interference is correctly accounted for in the simulations.

**Scenario 2—Analysis of D2D Mode-Switching Policies**

**Figure 21:**
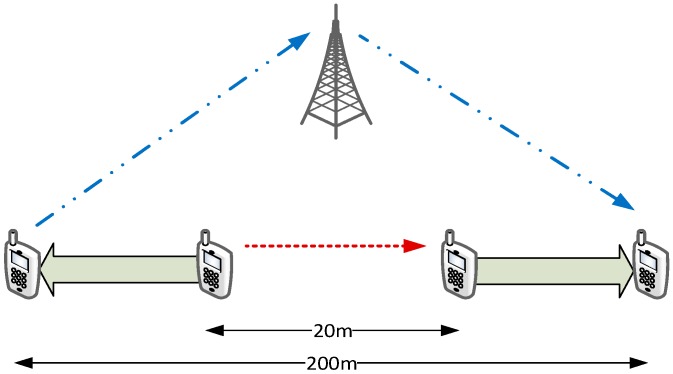
Simulation scenario for assessing the impact of MS.

**Figure 22:**
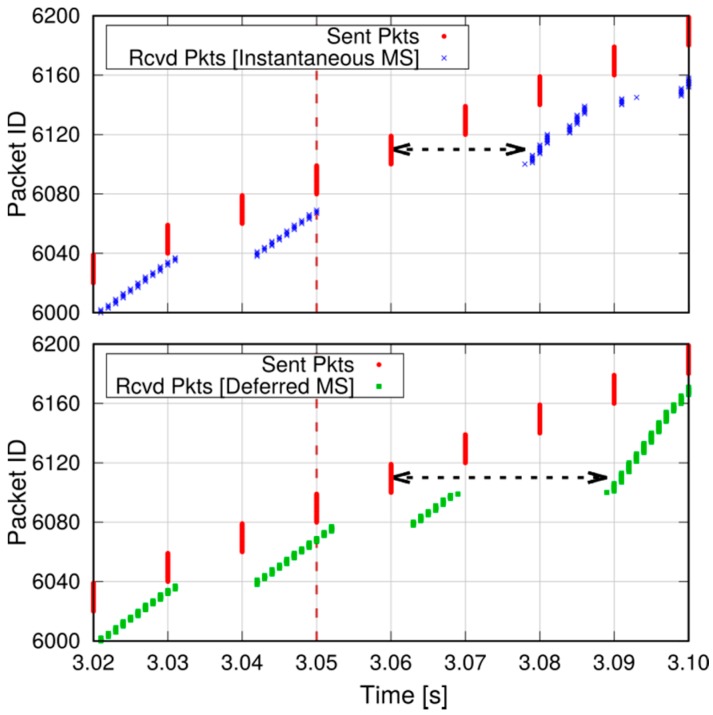
Effects of MS on a UDP-based application.

We now show the effects of different mode-switching (MS) schemes on the application-level performance of a D2D communication. With reference to the scenario in Figure 21, whose parameters are summarized in column *Scenario 2* of Table 4, we simulate a communication between two D2D-capable UEs. The initial distance between the two UEs is 20 m, and they start their communications on the SL, denoted by the dashed arrow. During the simulation, they move in opposite directions until they reach a distance of 200 m, hence their SL CQI decreases. After about 3 s (marked by a vertical dashed line in the figures), the SL CQI drops below the UL one, which prompts the eNB to order a MS of the connection to the infrastructure path. We compare two MS policies on the UEs, which we call Instantaneous MS and Deferred MS: in the former, the content of the transmitter’s RLC buffer is discarded, and new IP packets are sent along the new path. In the latter, new IP packets are buffered and MS is deferred until all the transmitter’s RLC buffer has been transmitted on the SL. We assume that the transmitter sends a burst of 20 packets every 10 ms using UDP as transport-layer protocol. Figure 22 shows the sequence of packets sent and received at the application level with the above two schemes. In the first case, a burst of packets is lost but packets sent just after the MS are not affected. On the other hand, deferred MS allows all the packets to reach the destination at the cost of increasing the delay of packets sent after the MS (dashed arrow in the figure).

**Figure 23:**
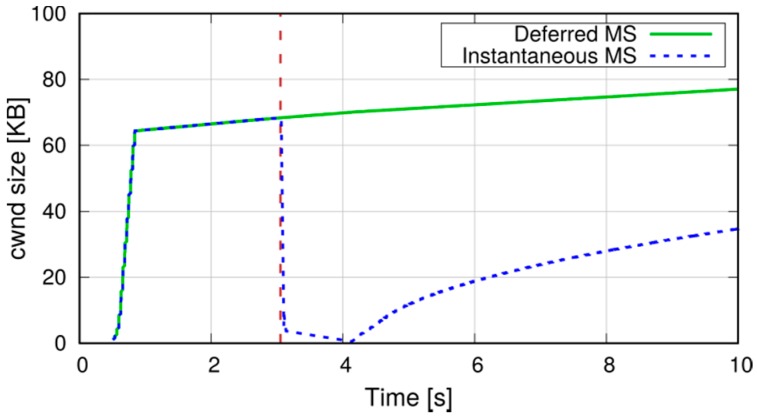
Congestion window of the TCP connection at a MS.

**Figure 24:**
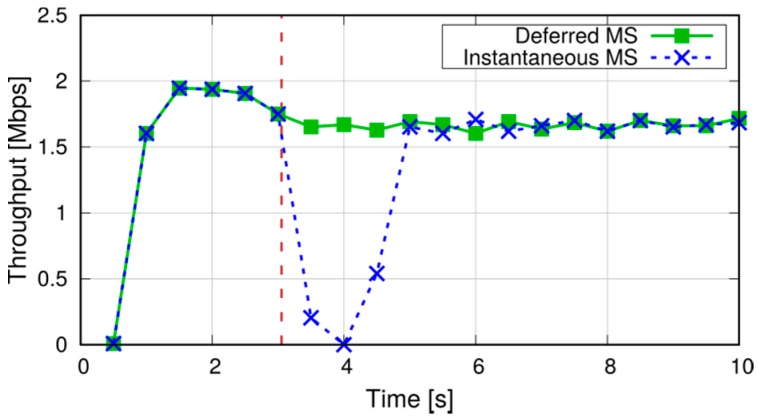
Application-level throughput of the TCP connection at a MS.

The effects of MS are amplified when TCP is used instead of UDP. Considering a file-transfer application in the same scenario as above, Figure 23 compares the evolution of the TCP congestion window with the two MS schemes. With the instantaneous MS, the discard of the RLC buffer triggers TCP congestion control: the sender receives duplicate acknowledgements and reduces its congestion window accordingly. Moreover, since the number of unacknowledged segments exceeds the new congestion window, the sender’s transmissions are halted until the retransmission timer expires (typically, after one second). With deferred MS, instead, the increased delay at the MS does not affect the congestion window (unless it exceeds the TCP timeout, which is not the case here). Figure 24 shows that the instantaneous application-level throughput is hardly affected in this case, its slight decrease being due to the larger round-trip time on the two-hop infrastructure path.

**Scenario 3—Selection of the P2MP transmission horizon**

**Figure 25:**
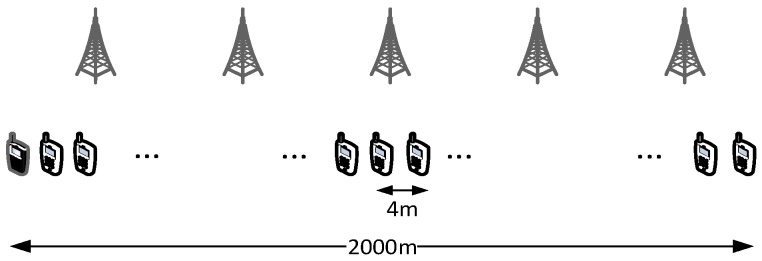
Simulation scenario for P2MP D2D transmissions.

**Figure 26:**
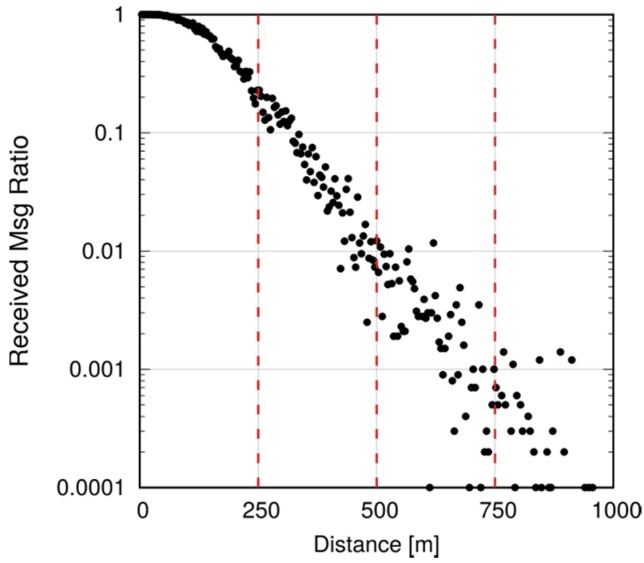
Reception probability of a P2MP D2D transmission.

We now evaluate the impact of setting a finite transmission horizon for P2MP D2D communications, according to the scenario in Figure 25 and the parameters in Table 4, *Scenario 3*. Five hundred UEs are placed four meters apart along a straight line, covering a distance of 2 km. Radio coverage is provided by five eNBs located at 400 m from each other. The leftmost UE sends 1000 largely spaced packets (one per second), using P2MP D2D transmissions. All UEs are members of the same multicast group. We recall that P2MP transmissions are modeled by invoking a sendDirect() for each potential receiver that is within a predefined distance. We initially use an infinite horizon, i.e., all the UEs in the network are added as targets of the sendDirect(). Every configuration is repeated ten times and the reported results are obtained as the average of such repetitions. Figure 26 reports the measured reception probability as a function of the distance from the transmitting UE, along with three vertical lines at 250, 500 and 750 m, representing possible values of the transmission horizon. Setting the horizon to 250 m excludes UEs that would receive with a probability of 20%. With *h =* 500 m, instead, we exclude UEs that would receive with 1% probability. With *h =* 750 m, only UEs with a reception probability around 0.1% are excluded.

**Figure 27:**
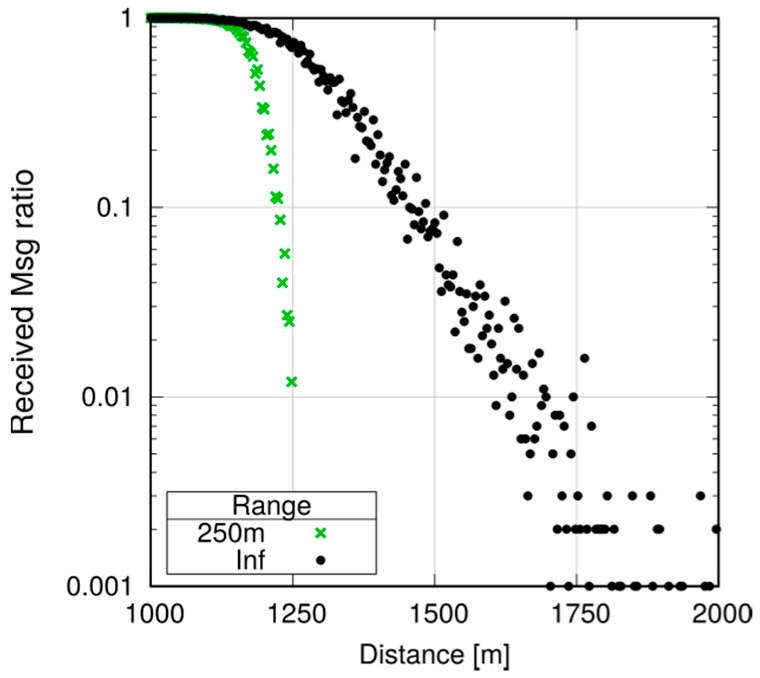
Reception probability in the multihop case, *h* = 250 m.

**Figure 28:**
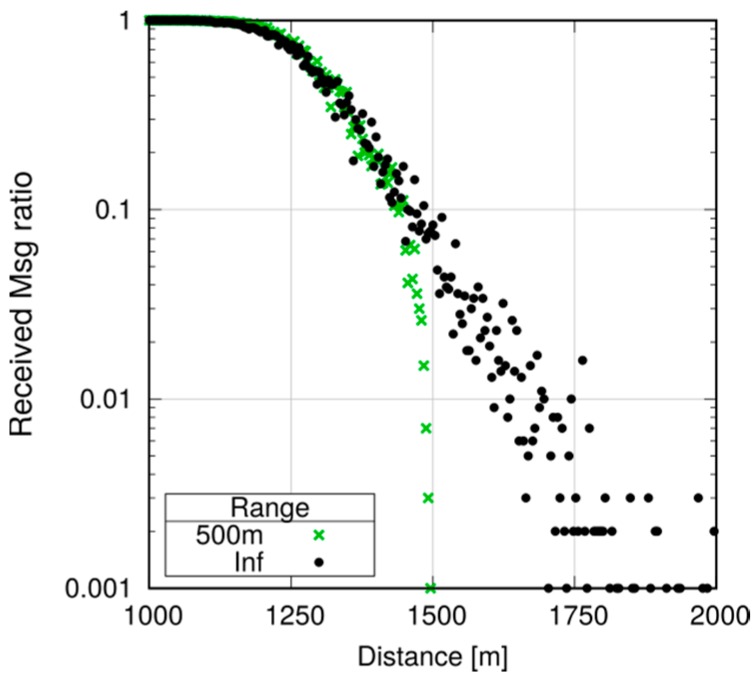
Reception probability in the multihop case, *h* = 500 m.

**Figure 29:**
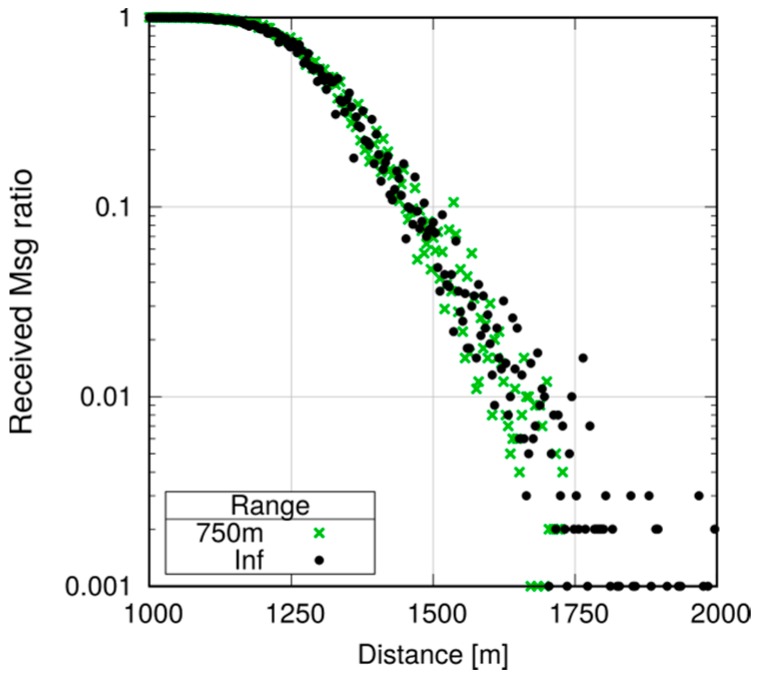
Reception probability in the multihop case, *h* = 750 m.

**Figure 30:**
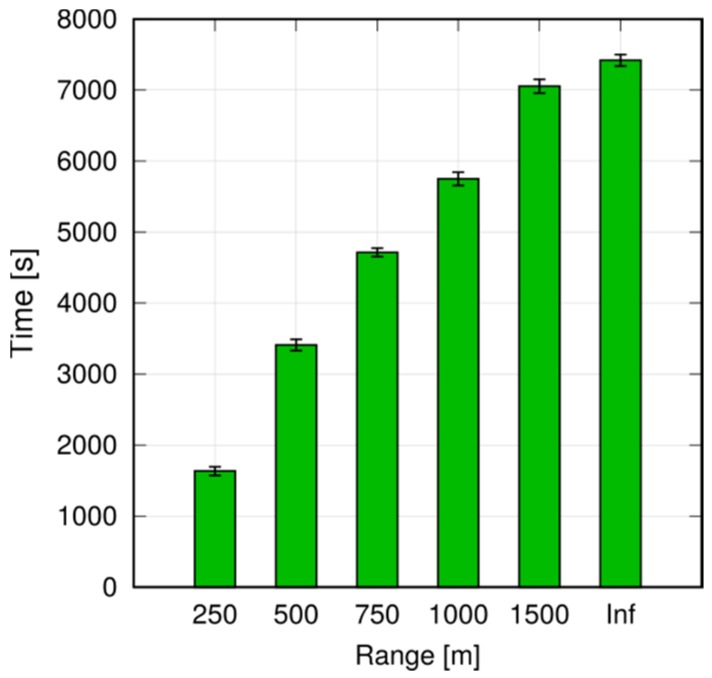
Simulation time.

Note, however, that even small differences may be amplified in a multihop scenario. Assume now that we want to ensure that every UE in the first 1000 m receives a message, and program all UEs to relay the message (still using P2MP D2D transmissions) if they are within 1000 m of the originator. To avoid excessive flooding, the Trickle duplicate suppression protocol is used, so that a UE that receives more than three copies of the message within a 10 ms time window assumes that the neighborhood is already well covered and suppresses relaying [6]. We test what happens by configuring a horizon of 250, 500 and 750 m for each transmission, compared to a baseline with an infinite horizon. Here, we run 10 repetitions with 100 s of simulated time. Figure 27, Figure 28 and Figure 29 show the probability of reception of UEs outside the 1000 m radius (those within the radius always receive with 100% probability), with a varying horizon *h*. Obviously, no UE located to the right of 1000 + *h* will receive any packet by definition, since no sendDirect() will target it. Figure 27 shows that setting *h =* 250 m completely overlooks the fact that UEs at, e.g., 1300 m, will receive nearly always in practice. Setting *h =* 500 m, as shown in Figure 28, overlooks UEs whose reception probability is around 10%, 10 times more than in the single-hop case. Finally, setting the horizon to 750 m overlooks UEs whose reception probability is 1%, as depicted in Figure 29. Figure 30 reports the simulation time as a function of the horizon *h*. We observe that the baseline employs just below 120 min, whereas introducing the transmission horizon abates the simulation time. In particular, the time is reduced by 37% when setting *h =* 750 m.

The take-home lesson is that limiting the transmission horizon may save a considerable amount of time in dense scenarios. However, the horizon should be set based on the expected residual reception probability, also accounting for multihop effects.

## 7. Conclusions and Future Work

In this paper, we have presented what—to the best of our knowledge—is the only model to date of network-controlled D2D communications in LTE-A networks. Our D2D model extends SimuLTE, a simulation library for LTE-A based on OMNeT++, compatible with the INET library. We have discussed the fundamental modeling choices underlying SimuLTE architecture, namely the split between logical and physical transmission, which allows us to include arbitrary interference models, without the burden of simulating broadcast air transmissions, as well as the modular framework for resource scheduling at the eNB. These modeling choices make it easy to incorporate new functionalities, namely D2D communications, into SimuLTE. We have shown that protocol layers for D2D-enabled UEs can be obtained via module inheritance. D2D-enabled resource allocation requires instead generalizing the one for standard cellular communication to allow for frequency reuse, which entails ownership of the same RBs by more than one transmitting UE at a time on the UL spectrum. We have described the data structures that we employ at the eNB to constrain frequency reuse, which are designed to minimize the complexity of checking for conflicts. We have shown how to limit the computational burden of broadcast P2MP transmissions, by using multicast groups and transmission horizons to limit the number of logical transmissions, while still computing the interference correctly.

Our profiling shows that D2D communications are not significantly costlier in terms of simulation time. On the one hand, single-hop transmission costs less than relaying at the eNB. On the other hand, computation and storage of SL CQIs (which are additional to UL CQIs) do add some overhead. Factorial analysis shows that the simulation duration is mainly determined by the number of UEs, whereas the overall traffic volume in the cell provides a negligible contribution, also in a D2D scenario. We have shown that limiting the transmission horizon for P2MP transmissions may warrant considerable reductions in simulation time, and that a reasonable trade-off between accuracy and simulation time can be reached. Moreover, we have shown the effects of different mode switching policies on the higher layers of the communication stack, and demonstrated that, even though our support for reuse-aware scheduling of D2D connections employs a binary interference model, interference is always correctly accounted for.

The ultimate purpose of releasing an open-source simulation library that models network-controlled D2D communications is to foster further research. More specifically, applications using D2D communications can now be prototyped and tested in a simulative environment. Their performance can be evaluated from both the user perspective—i.e., what performance the latter can expect from the underlying LTE-A network—and the network operator’s—i.e., how many resources they consume, and how to engineer the network to support them. Burgeoning fields for applications exploiting D2D are Vehicular Networks and Internet of Things. Moreover, our work allows competing schemes (e.g., for resource allocation) to be evaluated fairly and comprehensively, factoring in the (often counterintuitive) combination of effects of all the protocols in the LTE stack. For instance, it is actually the interplay of ciphering and numbering (at the PDCP) and buffering (at the RLC) that makes mode-switching a risky operation, where data loss may happen. Therefore, when designing mode-selection algorithms, the benefits of frequent mode selection should be assessed together with the potential switching-induced losses. Our simulation library is—to the best of our knowledge—the only existing tool that allows all of the above.

Our future work on this topic will involve developing a model for network-unassisted D2D communications.

## Figures and Tables

**Table 1:** Previous work describing existing LTE-A simulators and their D2D capabilities.

Reference	Focus	D2D Capabilities
[22]	Link level	No
[8]	Link level	Yes
[23]	Link level	No
[24]	Link level	Cooperation service among vehicles only
[25]	Link level	D2D Discovery only
[26]	Link level	Yes
[15]	System level	No
[14]	System level	No
[16]	System level	Network-unassisted D2D

**Table 2:** Parameter range for factorial analysis.

Name	Min	Max
**#UE pairs**	10	50
**System packet rate**	100 Pkt/s	500 Pkt/s

**Table 3:** Factorial analysis for execution time.

**Base Value**	431.45825	
**95% Conf. Int.**	±3.41	
	**Relative**	**Absolute**
**#UE-pairs**	97.98%	255.18
**System packet rate**	2.01%	36.58
**#UE-pairs x System packet rate**	0.005%	−1.77
**Unexplained**	0.001%	-

**Table 4:** Summary of main simulation parameters.

Parameter	Scenario 1 Selection of the Interference Radius	Scenario 2 Analysis of D2D Mode-Switching Policies	Scenario 3 Selection of the P2MP Transmission Horizon
**D2D comm. type**	P2P	P2P	P2MP
**Number of UEs**	2+[8,16,24,32] interferers	2	500
**Traffic pattern**	Constant Bit Rate (UDP-based)	Constant Bit Rate (UDP-based); 1 GB-file transfer (TCP-based)	Constant Bit Rate (UDP-based)
**UEs’ mobility**	Static	Linear (10 m/s)	Static
**UEs’ TX power**	15 dBm	15 dBm	15 dBm
**Simulation time**	30 s	30 s	100 s
**Take-home lesson**	Interference correctly accounted for, despite binary interference model	Application layer performance is affected by ineffective mode-switching policies	Reducing the transmission horizon abates simulation time
